# The absorption-addiction model of celebrity worship: in search of a broader theoretical foundation

**DOI:** 10.1186/s40359-024-01733-6

**Published:** 2024-04-23

**Authors:** Mara S. Aruguete, Frederick Grieve, Ágnes Zsila, Rita Horváth, Zsolt Demetrovics, Lynn E. McCutcheon

**Affiliations:** 1 Lincoln University, Missouri, MO USA; 2 Western Kentucky University, Bowling Green, KY USA; 3 Institute of Psychology, Pázmány Péter Catholic University, Budapest, Hungary; 4 Institute of Psychology, ELTE Eötvös Loránd University, Budapest, Hungary; 5 Doctoral School of Psychology, ELTE Eötvös Loránd University, Budapest, Hungary; 6 Centre of Excellence in Responsible Gaming, University of Gibraltar, Gibraltar, Gibraltar; 7 College of Education, Psychology and Social Work, Flinders University, Adelaide, Australia; 8 North American Journal of Psychology, Winter Garden, FL USA

**Keywords:** Celebrity worship, Empty self, Extremism, Passion

## Abstract

**Background:**

A large body of evidence suggests that some people have a strong, obsessive attachment to a favorite celebrity. The absorption-addiction model attempts to account for this extreme attachment, sometimes labeled “celebrity worship.” According to the model, a small portion of celebrity admirers (“celebrity worshipers”) become absorbed in the personal lives of celebrities to compensate for perceived personal or social deficiencies. The purpose of this study is to examine how the absorption-addiction model relates to broader psychological theories that include non-celebrity contexts. Specifically, we examine how the absorption-addiction model relates to three theories: empty-self theory, extremism theory, and the dualistic model of passion.

**Methods:**

Participants (*N* = 399; 77.94% women, *M*_*age*_ = 19.91 years, *SD* = 3.24) completed an online questionnaire measuring attraction to favorite celebrities. Constructs representing the three broader theories were compared to a measure of attraction to one’s favorite celebrity.

**Results:**

Sense of emptiness, obsessive passion, and extremism were positively associated with celebrity attraction. The strongest association was found with extremism, though the effect was moderate.

**Conclusions:**

These findings suggest that extremism theory is the best fit of the three broader theories explaining celebrity worship, although its contribution to understanding celebrity worship is modest.

**Supplementary Information:**

The online version contains supplementary material available at 10.1186/s40359-024-01733-6.

## Background

Celebrity worship can be defined as excessive interest in the lives of famous people [1]. A pattern of research findings over two decades has linked celebrity worship with psychologically unhealthy attitudes and behaviors [see 2 for a review]. The association between celebrity worship and maladaptive behaviors has been explained using the absorption-addiction model. The model posits that, while celebrity admiration exists on a continuum, some people, the “celebrity worshipers,” are intensely attracted to celebrities, idolizing them and following their every move. Their behaviors (e.g., searching the Internet for celebrity information) may take on addictive qualities, in which they become obsessed with the lives of celebrity figures [3]. However, the absorption-addiction model is relatively narrow in scope, explaining mostly extreme attraction to celebrities. The present study attempts to contextualize the absorption-addiction model with three similar theoretical models: empty-self theory, extremism theory, and the dualistic model of passion. Eronen and Bringmann [4] argue that the field of psychology has placed more emphasis on the development of statistical techniques than on theory building. Consequently, new theories are often developed to explain a narrow range of empirical findings with little attention to how they relate to extant theoretical explanation. This investigation addresses the question of whether the absorption-addiction model overlaps with similar theories that explain obsessive devotion. In particular, the current study attempts to link the absorption-addiction model to theories that emphasize the deleterious effects of excessive devotion to an idea, person or thing, especially as compensation for one’s psychological problems [3]. The three theories chosen shared features in common with the absorption-addiction model, based on research. Specifically, excessive gambling has been associated with both obsessive passion [5] and the absorption-addiction model [6]. Reeves et al. [7] also provided support for the convergence of correlates of celebrity worship and the empty-self theory.

### Absorption-addiction model of celebrity worship

The absorption-addiction model of celebrity worship stems from the work of McCutcheon et al. [1, 3, 8–11], who posited that extreme celebrity worship could be conceptualized as extreme admiration for a favorite celebrity, motivated by an attempt to compensate for psychosocial difficulties. Factor analysis of the Celebrity Attitudes Scale (CAS) revealed three levels of celebrity attraction. Level one (i.e., entertainment-social) is characterized as interest in a favorite celebrity primarily because of entertainment and social reasons. The favorite celebrity is often an entertainer whose accomplishments provide a topic that can be discussed with like-minded admirers. This first level is relatively benign. However, some admirers become obsessed and even addicted to their favorite celebrity, wanting to know intensely personal information (level two, intense-personal) and becoming willing to commit illegal or immoral acts to please that favorite celebrity (level three, borderline-pathological). The model is based on evidence showing that high scores on levels two and three of the CAS are positively associated with a range of psychosocial problems [2, 10, 12–14].

Many studies have supported the absorption-addiction model [3] showing that intense levels of celebrity admiration are associated with a range of maladaptive psychological states. Scores on the CAS have been positively related to depression, anxiety [15], neuroticism [12], poor relationship quality [16] compulsive buying and materialism [7, 17, 18], body dysmorphia [19], eating disorders [20], acceptance of stalking behaviors [16, 21], maladaptive daydreaming [22], and impulsivity [23]. Additionally, celebrity worship is negatively related to responsible attitudes [14]. Collectively, the research on extreme celebrity worship shows that it tends to be related to poor mental health, and that celebrity worship may serve as a compensation for deficiencies in one’s personal life (see [2] for a review).

### Empty-self theory

Empty-self theory has the potential to explain why some people worship celebrities. Cushman [24] introduced empty-self theory to explain dramatic changes in American society that occurred after the Second World War. In his view, the prevailing cultural trend was toward a “loss of community, tradition, and shared meaning,” and an increase in individualistic pursuits [24]. These absences have resulted in chronic emotional hunger (the empty self) that is reflected in an increase in narcissism, depression, materialism, compulsive consuming, difficulty in maintaining personal relationships, and overindulging hedonistic impulses [24]. We have become, in Cushman’s view, a nation of empty persons, struggling to temporarily fulfill ourselves with compulsively consumed goods, experiences, romantic partners, politicians who offer soothing but superficial solutions, and celebrities who are regarded as heroes. The post-World War II economy has supported the empty self by producing a continuous supply of non-essential goods and services accompanied by advertising designed to convince buyers that these goods and services are essential.

Support for the empty-self theory stems from studies that have found positive associations between materialism and compulsive buying to the negative outcomes that Cushman [24] identified, namely depression, narcissism, domestic dissension, low life satisfaction, anxiety, and poor psychological adjustment [23, 25–29].

Using materialism, compulsive buying, life satisfaction, boredom proneness and self-esteem as proxies for the empty self, Reeves et al. [7] found that celebrity worship was positively associated with all of them in ways consistent with empty-self theory. Furthermore, studies [17, 29] have also found associations between celebrity worship and materialism. Therefore, empty-self theory can be expected to contribute to the explanation of the absorption-addiction model of celebrity worship, insofar as parasocial relationships with celebrities may be one of many ways to compensate for personal feelings of emptiness.

### Dualistic model of passion

Passion is a strong positive feeling toward a specific object, activity, concept, or person, that one loves or strongly likes, highly values, invests time and energy in, and incorporates into one’s identity [30–32]. The dualistic model of passion [31] is so named because it argues for two types of passion: obsessive and harmonious. With obsessive passion, people experience a powerful and uncontrollable urge to engage with the favored activity/person/object, often at the expense of other activities in their lives. Obsessive passion comes with a price. If one becomes so obsessed with a favored activity, one may neglect other important activities. Such dogged persistence may lead to the achievement of superior performance, but it may also lead to life conflicts and other negative affective, cognitive, and behavioral consequences [32–34].

On the other hand, with harmonious passion, the favored activity/person/object occupies a significant but not overpowering portion of a person’s identity and is internalized in harmony with other important aspects of a person’s life. People engage in the favored activity willingly and flexibly. With harmonious passion, the person can momentarily drop the favored activity and engage in other activities when needed [31].

There is an extensive body of research showing that, whereas harmonious passion leads to relatively benign outcomes, obsessive passion leads to various forms of undesirable behaviors, including addiction, burnout, poor physical and mental health, and extreme interpersonal behaviors such as political and cause-related activism, stalking, and sports fanatic behavior [31, 34]. Conversely, harmonious passion is related to positive emotions and more adaptive outcomes such as well-being, positive relationship quality, less job burnout, and moderate and flexible involvement in an activity [29, 30, 33–35]. Moreover, Vallerand et al. [32] showed that harmonious passion was associated with positive emotions, whereas obsessive passion was associated with negative emotions.

There is reason to believe that the absorption-addiction model of celebrity worship would fit within the dualistic theory of passion. Though the dualistic model of passion explains a broad array of potential interests (in persons, activities, and objects), an obsessive passion for a favorite celebrity may share similarities with the construct of celebrity worship. In this sense, celebrity worship, as defined by the absorption-addiction model, could be a context-specific example of obsessive passion. According to Billieux et al. [36], obsessive passion can be viewed as an excessive engagement with an activity that can lead to loss of control over that activity, which can eventually interfere with other life domains (e.g., work, social relationships). Loss of self-control and impairment with daily life activities are core components of behavioral addictions (e.g., problematic Internet use, compulsive buying, gambling disorder), which have been associated with celebrity worship [6, 7, 22]. Therefore, we expect that those with a higher tendency to develop obsessive passion are also more prone to engage in excessive celebrity admiration.

### Extremism theory

Extremism can characterize a wide variety of attitudes and behaviors. This includes violent extremism [37], extreme infatuations, extreme dieting, extreme sports, extreme passions, addictions, and extreme dedication to a cause. A recent theory [38] argues that these attitudes and behaviors originate from the same psychological dynamic based on a motivational imbalance whereby a given need (or a goal serving this need) becomes dominant and overwhelms other basic needs. Virtually any kind of motivational imbalance can lead to extremism. Some extreme behaviors are viewed in a positive light by the public (e.g., the highly dedicated athlete who trains extremely hard and becomes a champion), but extremism often has negative consequences [38]. Highly dedicated athletes often get injured; besides, according to extremism theory, they become neglectful of other important needs that cause them problems. Increased commitment to one goal releases behavioral constraints. For instance, people who are not typically violent might consider violence to achieve their dominant goal, and people who are typically law-abiding can consider behaviors that are morally objectionable or even illegal if obsessed with their favorite celebrity [39].

Some people appear to be more prone to the motivational imbalance that characterizes extremism than others. That is, some people may exhibit a state of imbalance more often than others, and for some, the imbalance may last longer, and may push these individuals toward extreme attitudes and behaviors. Kruglanski et al. [38] refers to this as the “extreme personality.” For example, persons who are extreme celebrity worshipers have generally been shown to be extremely attached to their favorite celebrity over a three-month interval [9].

There is evidence to support the idea that the absorption-addiction model of celebrity worship fits under the umbrella of extremism theory. Similar to the dualistic model of passion [31], extremism theory [38] explains a broad array of potentially extreme devotions. Since the absorption-addiction model explains extreme interests in celebrities, it could be viewed as a context-specific manifestation of extremism within the context of the extremism theory. As noted above, persons who are highly absorbed or addicted to their favorite celebrity often show signs of poor psychological adjustment in a variety of ways. One possible interpretation of this poor adjustment is that persons who become extremely attached to their favorite celebrity show an imbalance in needs, becoming increasingly neglectful of intimate relationships [16] and exhibiting irresponsible attitudes [14], such as condoning the stalking of celebrities [21, 39], resulting in lower satisfaction with life [23]. Therefore, we expect that extremism contributes to the explanation of celebrity worship.

### Hypotheses

We hypothesize that feelings of emptiness, obsessive (but not harmonious) passion, and extremism will be positively related to celebrity worship. We also examine the explanatory power of these constructs in celebrity worship. To compare the predictive power of these constructs, we explore the contribution of each factor to celebrity worship separately. Subsequently, we construct a structural equation (SEM) model that includes all hypothetical predictors. Results will allow us to determine the extent to which each of the three theories can explain the absorption-addiction model of celebrity worship.

## Methods

### Participants

We recruited 438 participants from three universities located in the United States. Students were recruited by posting announcements on course websites and on a web-based university participant recruitment system. Approximately 987 students had access to the survey (44% response rate). Exclusion criteria were missing demographics (*n* = 17), two or more measures incomplete (*n* = 14), and no favorite celebrity named (*n* = 8). Our final sample consisted of 399 undergraduate students (77.94% women, *M*_*age*_ = 19.91 years, *SD* = 3.24, range: 18–47 years of age). Most participants ethnically identified as White (*n* = 292, 73.18%), followed by Black (*n* = 51, 12.78%), Latinx (*n* = 22, 5.51%), and Asian-American (*n* = 13, 3.26%). A smaller proportion selected ‘other’ ethnic group (*n* = 21; 5.26%).

#### Ethical approval

Ethical approval was obtained from Institutional Review Boards of Lincoln University and Western Kentucky University (IRB# 22–273). The research protocol was in accordance with the Declaration of Helsinki. Participants provided informed consent. Participation in the study was voluntary and anonymous. Course credit was awarded to each participant.

### Procedure

Participants completed an online questionnaire measuring celebrity worship, emptiness, passion, and extremism in random order to reduce the likelihood of a systematic order effect [40]. An a priori power analysis using GPower [41] indicated that a total sample size of 189 would be needed to detect a small-to-moderate effect size of *f*^*2*^ = 0.075 [42] with 80% power using OLS multiple regression (fixed model, deviation from zero) with alpha at 0.05 (effect size conventions for *f*^2^ are 0.02 = small, 0.15 = moderate, 0.35 = large effect [42]). This estimation was based on previous findings indicating weak and weak-to-moderate associations between celebrity worship and materialism, compulsive buying [7], stalking behaviors [21], and irresponsible attitudes [14].

### Measures

#### Celebrity attitude scale

The 23-item *Celebrity Attitude Scale* (*CAS*) was used to measure celebrity worship. The scale has shown good psychometric properties across a range of samples [3, 9, 11, 12, 22, 43–45]. We used a version of the CAS in which 10 of the 23 items were reverse-scored, called the CAS-D. Sample items include “My friends and I like to discuss what my favorite celebrity has done,” and “When something good happens to my favorite celebrity, I don’t feel like it happened to me” (reversed), The response options for the *CAS* range from 1 (*strongly disagree*) to 5 (*strongly agree*). High scores indicate stronger worship of a favorite celebrity. Across several studies, the total scale Cronbach’s alpha values ranged from 0.84 to 0.94 [1, 6]. Reliability indices for the present study are presented in Table 1.

#### Multidimensional sense of emptiness scale (MSES)

The 16-item MSES was used, developed and validated by Ermis-Demirtas [46]. Examples of items on each of the four subscales include: Sense of Inner Emptiness (MSES Emp) – “I feel emotionally hungry”; Sense of Meaninglessness (MSES Mean) – “My life has no clear direction”; Sense of Absence of Relatedness (MSES Relate) – “I feel I am not relating to anyone”; and Sense of Spiritual Emptiness (MSES Spirit) – “I feel distant from my Higher Power/Divine/God”. Respondents are asked to read each statement and determine how often the statement is generally true for them over the last year. The scale ranges from 0 (*none of the time true of me*) to 6 (*all of the time true of me*). High scores indicate feeling empty inside, in accordance with empty-self theory [24]. An alpha coefficient of 0.98 was found for the full version of the MSES in the original study [46].

#### Passion scale

To assess harmonious and obsessive passion, we used six items of the *Passion Scale* [PS; 31]. Items were selected prior to data collection based on high factor loadings derived from Vallerand et al. [31]. Participants were first asked to think of an activity that is very dear to their hearts. Then, with this activity in mind, they rated their agreement to the six passion items. A sample item for obsessive passion was “I have almost an obsessive feeling toward this activity.” A sample item for harmonious passion was “This activity is in harmony with other activities in my life.” Participants responded on a scale from 1 (*do not agree at all*) to 7 (*completely agree*). There were three items per type of passion. High scores on each subscale suggest persons who are passionate about their favorite activity in a harmonious or obsessive way. In previous research, Cronbach’s alphas for the harmonious passion subscale were 0.72 and 0.78; for obsessive passion, alphas were 0.83 and 0.86 [30].

##### Extremism scale

The *Extremism Scale* (ES) was developed to test the theory of extremism [47]. This 12-item scale uses a Likert-type format anchored by 1 (*definitely disagree*) to 7 (*definitely agree*). Sample items include “When I decide on something, I go for it like my life depended on it” and “I react very emotionally to anything that is related to my most important goal.” Item 10 was inserted as an attention check. Test-retest reliability with a 4- to 10-week interval was 0.74. Cronbach’s alphas across 18 separate studies ranged from 0.84 to 0.95 [47].

### Statistical analysis

First, correlation analysis was conducted using SPSS 21.0. As the skewness and kurtosis was within the range of -2 and + 2 for all variables, data were considered approximately normally distributed according to George and Mallery [48]. Therefore, Pearson correlations were performed to explore the associations between variables.

Second, multiple regression analyses with latent variables were conducted using Mplus 7.4 [49]. All models were conducted using a robust weight least square estimator with mean and variance adjusted statistics (WLSMV), which is appropriate for categorical variables [50]. According to the Mardia test [51], multivariate normality was not supported either for skewness (*p* < 0.001), or kurtosis (*p* < 0.001) for the full model.

Listwise deletion was used to handle missing data, which is the default when using a WLSMV estimator. Therefore, regression models were estimated using *n* = 387 cases. The following model fit indices were applied [52, 53]: the Comparative Fit Index (CFI; ≥ 0.90 for acceptable), the Tucker-Lewis Index (TLI; ≥ 0.90 for acceptable), and the root-mean-square error of approximation (RMSEA; ≤ 0.08 for acceptable) and its 90% confidence interval (90% CI). Models were estimated using 1,000 bootstrapped replication samples. To improve model fit, an error covariance was added between the second the third item of the extremism scale based on the modification indices. These two items had a severe overlap in their content and were highly correlated (*r* = 0.80). As some previous studies have indicated gender and age differences in celebrity worship [e.g., 2, 54, 55], these demographic variables were included in the models as covariates. Effect sizes for correlation (*r* = 0.10–0.29 as a small, *r* = 0.30–0.49 as a medium, and *r* ≥ 0.50 as a large effect) and *β* coefficients (*β* = 0.10–0.29 as a small, *β* = 0.30–0.49 as a medium, and *β* ≥ 0.50 as a large effect) were considered based on the thresholds provided by Cohen [42].

## Results

### Descriptive statistics

Participants selected mostly musicians (*n* = 135; 33.83%), actors (*n* = 118; 29.57%), and artists (*n* = 51; 12.78%) as favorite celebrities. A smaller proportion of participants selected ‘sports’ as a primary field of expertise of their favorite celebrity (*n* = 32; 8.02%), while another 15.79% (*n* = 63) selected other fields (e.g., modeling, politics). The most frequently selected favorite celebrities were Taylor Swift (*n* = 26; 6.51%), followed by Harry Styles (*n* = 17; 4.26%), and Zendaya Coleman (*n* = 10; 2.51%).

### Associations between celebrity worship, emptiness, passion, and extremism

Pearson correlations were conducted to explore the associations between variables (see Table 1). As hypothesized, celebrity worship was weakly, positively associated with emptiness and obsessive passion, but not with harmonious passion. Celebrity worship was also positively associated with extremism, with a weak-to-moderate effect size.

**Table 1:** **D**escriptive statistics and pearson correlations among all study variables

	1.	2.	3.	4.	5.	6.	7.	8.	9.
1. CAS Total	–								
2. MSES Total	0.20***	–							
3. MSES Emp	0.19***	0.83***	–						
4. MSES Meaning	0.14**	0.86***	0.66***	–					
5. MSES Relate	0.10	0.80***	0.59***	0.64***	–				
6. MSES Spirit	0.20***	0.75***	0.47***	0.48***	0.41***	–			
7. HP	0.03	-0.09	-0.04	-0.10*	-0.13*	-0.02	–		
8. OP	0.24***	0.24***	0.22***	0.22***	0.18***	0.17**	0.14**	–	
9. Extremism	0.36***	0.28***	0.27***	0.17**	0.30***	0.19***	0.05	0.32**	–
Range	25–99	0–96	0–24	0–24	0–24	0–24	1–7	1–7	12–84
Mean	60.12	24.89	8.11	5.37	6.09	5.33	5.50	3.25	48.80
SD	13.63	20.10	6.05	6.75	5.82	6.25	1.12	1.58	13.79
Skewness	0.01	0.96	0.62	1.32	1.00	1.33	-1.05	0.33	-0.16
Kurtosis	-0.34	0.48	-0.41	0.98	0.25	0.68	1.96	-0.71	-0.51
Cronbach’s α	0.88	0.94	0.87	0.94	0.88	0.92	0.83	0.84	0.90
McDonald ω	0.88	0.93	0.87	0.94	0.89	0.92	0.83	0.84	0.91

Note. ***p < 0.001; **p < 0.01; *p < 0.05

CAS = Celebrity Attitude Scale; MSES = Multidimensional Sense of Emptiness Scale; Emp = Sense of Inner Emptiness; Meaning = Sense of Meaninglessness; Relate = Sense of Absence of Relatedness; Spirit = Sense of Spiritual Emptiness; HP = Harmonious Passion; OP = Obsessive Passion; SD = Standard Deviation

The total number of observations was *N* = 397 for the MSES variables, *N* = 398 for HP and OP, and *N* = 399 for all other variables

### The explanatory power of emptiness, passion, and extremism in the absorption-addiction model of celebrity worship

First, we examined the explanatory power of variables separately in celebrity worship. Consistent with the results of the correlation analysis, feelings of emptiness, obsessive passion, and extremism were significant predictors of celebrity worship, but with weak explanatory power (see Table 2). The highest contribution to the explanation of celebrity worship was provided by extremism. Results regarding the three subscales of the CAS are presented in SM Table 1 for a more detailed overview.

**Table 2:** Multiple regression models predicting celebrity worship

Predictor variables	Outcome variable: celebrity worship	
	β (SE)	95% CI
**Model I**		
Gender	0.03 (0.06)	-0.07; 0.15
Age	-0.08 (0.05)	-0.17; 0.02
**R** ^**2**^	0%	
**Model II**		
Gender	0.02 (0.06)	-0.09; 0.14
Age	-0.09 (0.05)	-0.18; 0.01
Emptiness	0.26 (0.06)***	0.16; 0.37
**R** ^**2**^	8%	
**Model III**		
Gender	0.03 (0.06)	-0.08; 0.14
Age	-0.09 (0.05)	-0.18; 0.01
Harmonious passion	-0.02 (0.07)	-0.15; 0.11
Obsessive passion	0.32 (0.06)***	0.18; 0.43
**R** ^**2**^	11%	
**Model IV**		
Gender	0.02 (0.06)	-0.09; 0.14
Age	-0.09 (0.05)	-0.18; 0.01
Extremism	0.41 (0.05)***	0.31; 0.50
**R** ^**2**^	17%	

*Note. N* = 387. *** *p* < 0.001 95% confidence intervals are reported based on 1,000 bootstrapped samples

Gender (1 = *men*, 2 = *women*) and age were added as observed control variables in models II–IV

Celebrity worship, emptiness, the two types of passion, and extremism were latent variables. Model fit indices were adequate (*χ*^*2*^ = 10.06, *df* = 4, *p* = 0.04; CFI = 0.986; TLI = 0.969; RMSEA = 0.063 [90% CI = 0.013–0.112] for Model I; *χ*^*2*^ = 571.07, *df* = 183, *p* < 0.001; CFI = 0.978; TLI = 0.975; RMSEA = 0.074 [90% CI = 0.067–0.081] for Model II; *χ*^*2*^ = 89.61, *df* = 40, *p* < 0.001; CFI = 0.982; TLI = 0.976; RMSEA = 0.057 [90% CI = 0.041–0.072] for Model III; *χ*^*2*^ = 370.77, *df* = 116, *p* < 0.001; CFI = 0.962; TLI = 0.956; RMSEA = 0.075 [90% CI = 0.067–0.084] for Model IV.

**Fig. 1:**
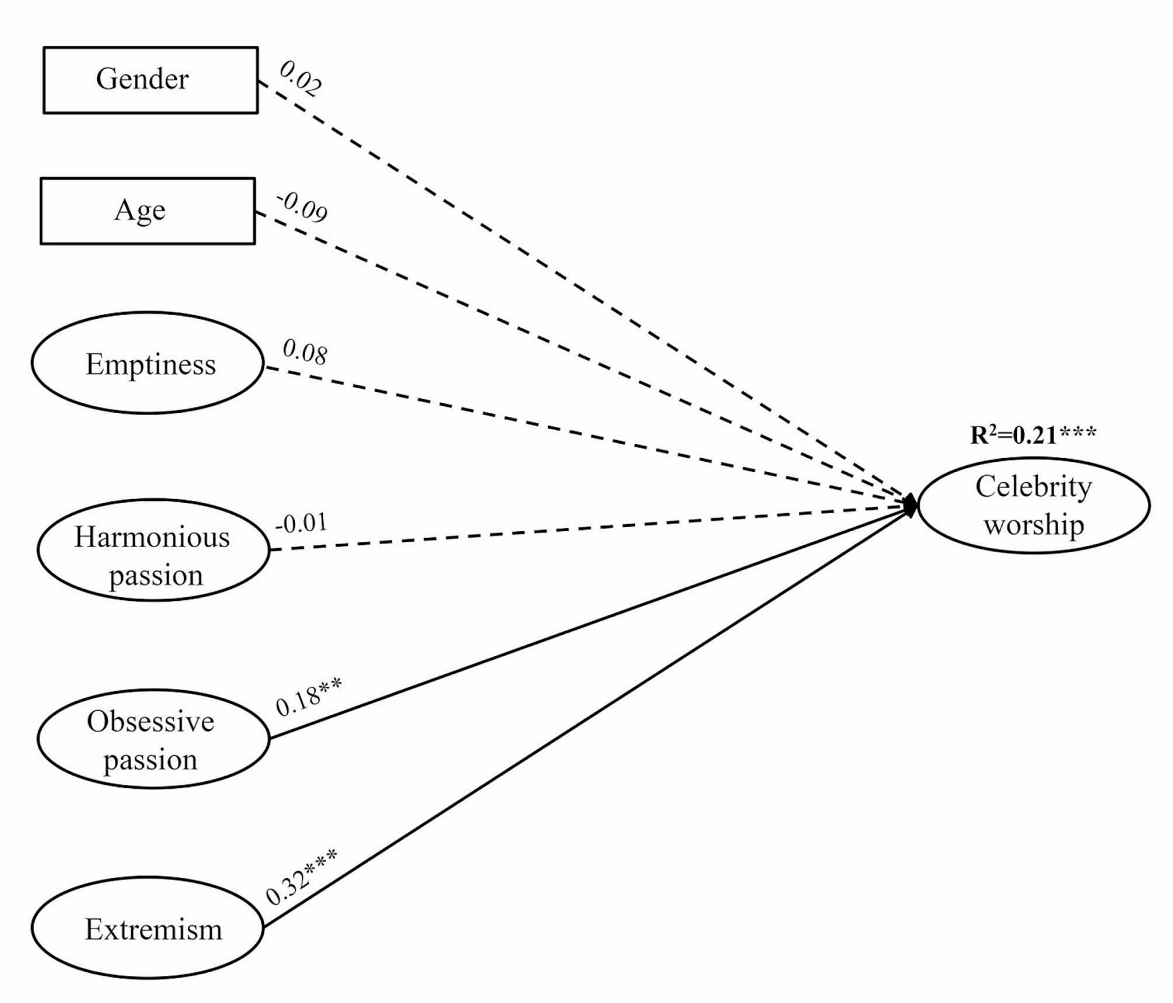
Multiple regression analysis with latent variables predicting celebrity worship. *Note.* *** *p* < 0.001; ** *p* = 0.01. *N* = 387. Gender (1 = *men*, 2 = *women*) and age were included as covariates in the model. Standardized regression coefficients (*β*s) are presented on the arrows. Nonsignificant associations are presented with dashed arrows. Ovals represent latent variables, and rectangles represent observed variables

In the next step, all possible explanatory variables were entered simultaneously in the multiple regression model to investigate the associations of variables from the three theories with celebrity worship in a single complex model (see Fig. 1). The SEM model fit was adequate (*χ*^*2*^ = 1257.44, *df* = 686, *p* < 0.001; CFI = 0.907; TLI = 0.900; RMSEA = 0.046 [90% CI = 0.042–0.050]). Extremism showed a positive, moderate association, while obsessive passion had a weak, positive association with celebrity worship. In this model, emptiness was not significantly associated with celebrity worship. These variables explained a small proportion of the total variance of celebrity worship (21%). Associations for the three subscales of the CAS are resented in SM Fig. 1 for a more detailed overview.

## Discussion

The present study was conducted to contextualize [4] the absorption-addiction model of celebrity worship [1, 3, 8–11] among existing theories. Three theories that were proposed to have explanatory power for the absorption-addiction model were the empty-self theory [24], the dualistic model of passion [31], and extremism theory [38]. We proposed that feelings of emptiness, obsessive (but not harmonious) passion, and extremism would all be positively related to celebrity worship, but the contribution to the explanation of celebrity worship may vary.

Correlation analysis supported this hypothesis. However, when exploring the explanatory power of these scales derived from the three theories, results showed that extremism and obsessive passion positively predicted celebrity worship, but emptiness did not. As predicted, harmonious passion was not associated with celebrity worship.

These findings may contribute to a more detailed picture of people who worship celebrities. They experience the need to follow or be a part of a celebrity’s life and this need dominates their lives. It is even possible that their identity is wrapped up in the idea of being a “superfan.” Such an extreme focus on the celebrity may lead to obsessive passion about the celebrity and a drive to participate in the parasocial behaviors that comprise celebrity worship. For these fans, celebrity worship consumes their lives and is the most important aspect of their lives. Such relationships support the idea that extreme celebrity worshipers have at least some characteristics of an “extreme personality” [38, 47]. These results support Billieux et al. [36] in that extreme celebrity worship involves excessive engagement in celebrity worship behaviors.

Pushing behaviors to extreme limits is not only found with celebrity worship. Researchers in other areas of fandom have noted extreme and maladaptive behaviors. For example, Wakefield and Wann [56] described “dysfunctional” sport fans with similar types of descriptors. Dysfunctional fans are complaining and confrontational; they display problematic behaviors at sporting events such as excessive consumption of alcohol and berating officials and fans of the opponent. Dysfunctional fandom has been associated with most inappropriate or maladaptive behaviors conducted by sport fans [57]. Similarly, Havard et al. [58] described a Hierarchy of Outgroup Derogation (HOD) that evaluates how much fans of different teams do not like each other. While this idea was first noticed in sport fans, the HOD can be applied to fans of politics, science fiction, video games, and comic books. Fans who display more negative behaviors, including fans of video games and politics, can be considered more extreme in their nature.

Our correlation analysis showed that emptiness was associated with celebrity worship, although this relationship was slightly weaker than the associations between obsessive passion and extremism with celebrity worship. However, when emptiness was included in a multiple regression with the other variables, it lost its ability to predict celebrity worship.

These findings are consistent with those of Reeves et al. [7] and Green et al. [17]. They found relationships between celebrity worship and materialism, which was used as a proxy for having an empty life. Thus, it might be that materialism is a poor representation of feeling emptiness. On the other hand, Green et al. [17] found a particularly weak association between these two constructs. Reeves et al. [7] also found only a weak predictive power of materialism in celebrity worship.

Overall, these findings allow for a broader contextualization of the absorption-addiction model of celebrity worship [4] that can extend the scope of previous empirical investigations. The absorption-addiction model appears to be congruent with extremism theory [38] and the dualistic model of passion [31], but the empty-self theory needs further investigation, especially in wealthy Western nations similar to the United States [24]. In addition, inter-relationships between extremism, emptiness and obsessive passion provide some further evidence of concurrent validity [59] for each of the measures of these theories. Since the observed relationships cannot be inferred as causal, extremism theory and the dualistic model of passion receive as much support for predictions made using those theories as does the absorption-addiction model of celebrity worship.

Results of the study indicated that participants selected musicians (33.83%), actors (29.57%), and artists (12.78%) as their favorite celebrities. These percentages are in line with other research that has indicated that musicians, actors, and athletes are the most popular choices. Choices of a favorite celebrity stem from one of these three categories for 70–80% of participants [1, 9, 22, 60, 61].

The study has some important limitations. The participants for this study were young college-educated students, mostly female, and mostly White. Thus, generalization of these findings to other populations is limited. Thirty-nine participants were excluded because of missing data. Due to the absence of demographic data in most of these cases, it was impossible to assess the extent to which this group differed from the group included in the analyses. Additionally, specific operational definitions (measures of each theory) were used for each of the theoretical constructs under study. Alternative measures of each theory might yield different results. The measure of passion in this study asked participants to base their responses on an activity “near and dear to their hearts”. Without having measured the specific activity, it was impossible to evaluate whether activities coincided with celebrity worship. If this were the case, the passion scale and the celebrity attitudes scale may have been highly correlated. Finally, the data were collected via self-report measures. Self-report measures are subject to several biases, including impression management [62].

Despite these limitations, the results suggest areas for future research. The next important step would be to investigate the temporal relationship between celebrity worship and extremism and obsessive passion. That is, does celebrity worship precede or postdate the characteristics of obsessive passion and extremism? Similarly, though all the theories discuss psychological problems (e.g., depression, anxiety) associated with extreme devotion, they differ in whether such problems are conceptualized as antecedents or consequences of the extreme devotion. Thus, longitudinal research in the area is warranted. A second area of investigation could be to evaluate the rivalry among extreme fans of different celebrities, similar to the extremism seen in sports fandom. It seems as though there would be high levels of outgroup derogation [58] between extreme fans of different celebrities (e.g., JoJo Siwa and Candace Cameron Bure [63]) who are in a feud. Classifying the derogation and mapping the relationships would be an interesting area of research.

Despite these limitations, the results suggest areas for future research. The next important step would be to investigate the temporal relationship between celebrity worship and extremism and obsessive passion. That is, does celebrity worship precede or postdate the characteristics of obsessive passion and extremism? Similarly, though all the theories discuss psychological problems (e.g., depression, anxiety) associated with extreme devotion, they differ in whether such problems are conceptualized as antecedents or consequences of the extreme devotion. Thus, longitudinal research in the area is warranted. A second area of investigation could be to evaluate the rivalry among extreme fans of different celebrities, similar to the extremism seen in sports fandom. It seems as though there would be high levels of outgroup derogation [58] between extreme fans of different celebrities (e.g., JoJo Siwa and Candace Cameron Bure [63]) who are in a feud. Classifying the derogation and mapping the relationships would be an interesting area of research.

In conclusion, results of the present study provide support for the relationship between the absorption-addiction model of celebrity worship and both extremism theory and the dualistic theory of passion. Specifically, high levels of celebrity worship were associated with higher levels of extremism and higher levels of obsessive passion, but were not related to harmonious passion. Extremism showed the greatest contribution to explain celebrity worship; however, its explanatory power was still modest. Future studies should broaden the scope of theoretical investigations to other theories such as the dual model of escapism [64].

### Electronic supplementary material

Below is the link to the electronic supplementary material.

Supplementary Material 1

## Data Availability

The datasets used and/or analyzed during the current study available from the first author on reasonable request.
