# Supplementary material for: The absorption-addiction model of celebrity worship: in search of a broader theoretical foundation
Source: BMC Psychol. 2024 Apr 23;12:224. doi: 10.1186/s40359-024-01733-6 (PMC11041038; doi:10.1186/s40359-024-01733-6)
Supplement: Supplementary file 1 — Supplementary Material 1 [file 40359_2024_1733_MOESM1_ESM.docx]

**SM Table 1**

*Multiple Indicators Multiple Causes (MIMIC) Model Predicting Celebrity Worship Dimensions*

| Predictor variables | Outcome variable: celebrity worship | | | | | |
| --- | --- | --- | --- | --- | --- | --- |
|  | Entertainment-social | | Intense-personal | | Borderline-pathological | |
|  | *β* (SE) | 95 % CI | *β* (SE) | 95 % CI | *β* (SE) | 95 % CI |
| **Model I** |  |  |  |  |  |  |
| Gender | 0.07 (0.05) | -0.02; 0.18 | -0.01 (0.05) | -0.11; 0.09 | 0.003 (0.05) | -0.11; 0.10 |
| Age | -0.08 (0.04) | -0.16; 0.003 | -0.12 (0.05)* | -0.21; -0.02 | -0.01 (0.04) | -0.09; 0.08 |
| **R^2^** | 0% |  | 0% |  | 0% |  |
| **Model II** |  |  |  |  |  |  |
| Gender | 0.07 (0.05) | -0.02; 0.18 | -0.01 (0.05) | -0.11; 0.09 | 0.003 (0.05) | -0.11; 0.10 |
| Age | -0.08 (0.04) | -0.16; 0.003 | -0.12 (0.05)* | -0.21; -0.02 | -0.01 (0.04) | -0.09; 0.08 |
| Emptiness | 0.21 (0.05)*** | 0.11; 0.32 | 0.18 (0.05)*** | 0.08; 0.28 | 0.23 (0.05)*** | 0.13; 0.32 |
| **R^2^** | 6% |  | 5% |  | 5% |  |
| **Model III** |  |  |  |  |  |  |
| Gender | 0.07 (0.05) | -0.02 ; 0.18 | -0.01 (0.05) | -0.11; 0.09 | 0.003 (0.05) | -0.11; 0.10 |
| Age | -0.08 (0.04) | -0.16; 0.003 | -0.12 (0.05)* | -0.21; -0.02 | -0.01 (0.04) | -0.09; 0.08 |
| Harmonious passion | 0.11 (0.06) | 0.01; 0.23 | -0.10 (0.06) | -0.21; 0.02 | -0.07 (0.06) | -0.19; 0.04 |
| Obsessive passion | 0.24 (0.06)*** | 0.12; 0.35 | 0.25 (0.06)*** | 0.12; 0.35 | 0.26 (0.06)*** | 0.15; 0.36 |
| **R^2^** | 9% |  | 8% |  | 7% |  |
| **Model IV** |  |  |  |  |  |  |
| Gender | 0.07 (0.05) | -0.02 ; 0.18 | -0.01 (0.05) | -0.11; 0.09 | 0.003 (0.05) | -0.10; 0.10 |
| Age | -0.08 (0.04) | -0.16; 0.003 | -0.12 (0.05)* | -0.21; -0.02 | -0.01 (0.04) | -0.09; 0.08 |
| Extremism | 0.30 (0.05)*** | 0.20; 0.39 | 0.35 (0.04)*** | 0.25; 0.43 | 0.32 (0.05)*** | 0.22; 0.40 |
| **R^2^** | 10% |  | 13% |  | 10% |  |

*Note.*

*N*= 387.

*** *p* < 0.001; * *p* < 0.05

The three dimensions of celebrity worship were entered simultaneously in the MIMIC model as observed variables, while emptiness, the two types of passion, and extremism were latent variables.

Gender is coded as 1 = *men*, 2 = *women*.

95% confidence intervals are reported based on 1,000 bootstrapped samples.

Gender and age were added as covariates to models II–IV.

The internal consistency of the borderline-pathological subscale of the Celebrity Attitudes Scale was low (α = 0.45, ω = 0.48); therefore, results relating to this subscale should be interpreted with caution.

**SM Figure 1**

*Multiple Indicators Multiple Causes (MIMIC) Model Predicting the Three Dimensions of Celebrity Worship*

*
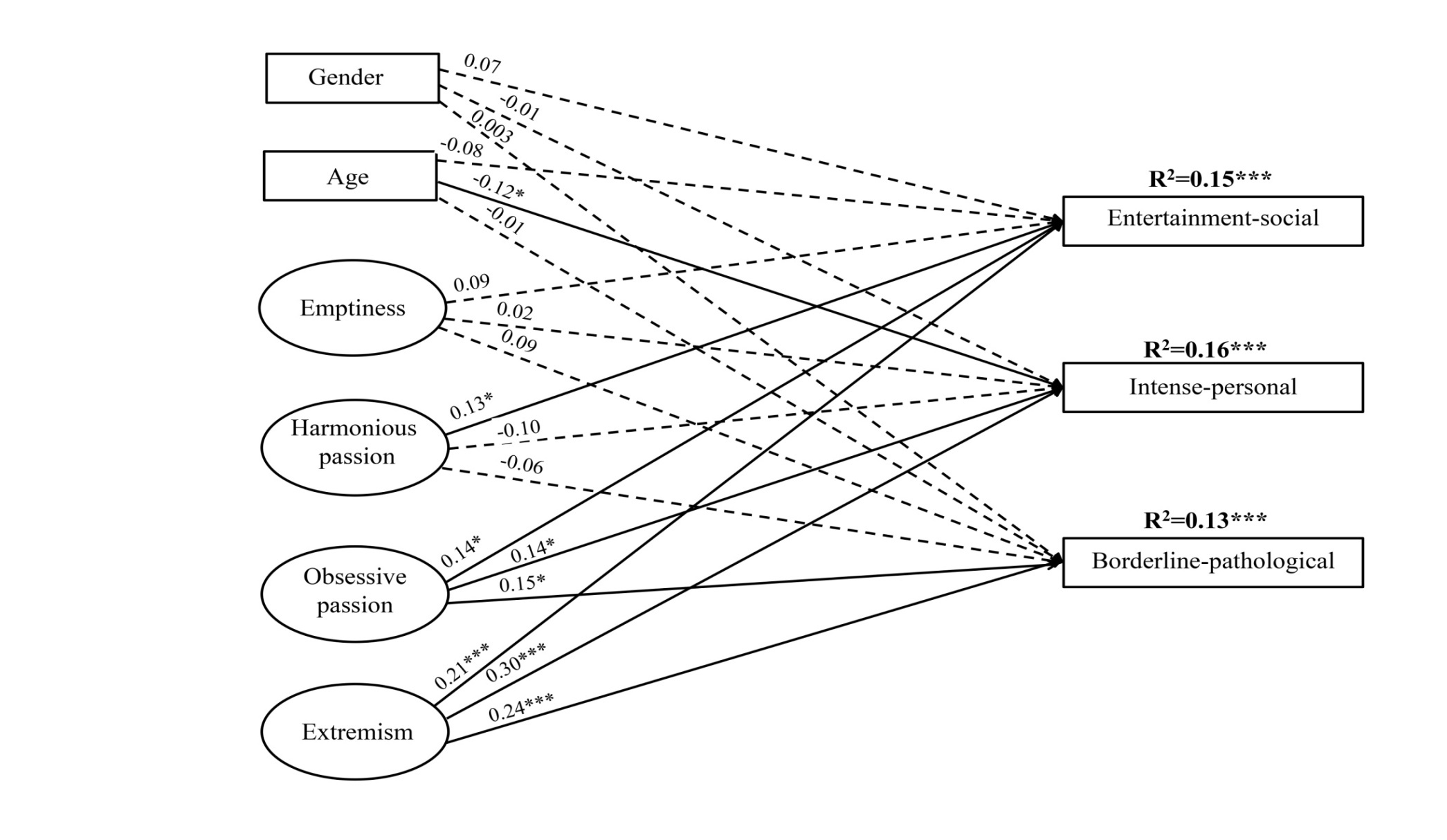
*

*Note.*

*** *p* < 0.001; * *p* < 0.05

*N*= 387.

Gender (1 = *men*, 2 = *women*) were included as covariates.

Standardized regression coefficients (*β*s) are presented on the arrows.

Nonsignificant associations are presented with dashed arrows.

Ovals represent latent variables, and rectangles represent observed variables.

The internal consistency of the borderline-pathological subscale of the Celebrity Attitudes Scale was low; therefore, results relating to this dimension should be interpreted with caution.

Model fit indices indicated an acceptable fit (*χ^2^* = 1234.48, *df* = 674, *p* < 0.001; CFI = 0.909; TLI = 0.900; RMSEA = 0.046 [0.042–0.050]).
